# How Do Similarity Cues Shape Children’s Social Preference Inferences?

**DOI:** 10.3390/bs16071121

**Published:** 2026-07-04

**Authors:** Youjung Choi, Alyssa M. Cooley

**Affiliations:** School of Psychological and Behavioral Sciences, Southern Illinois University, Carbondale, IL 62901, USA; alyssa.cooley@siu.edu

**Keywords:** social preference, similarity cues, social cognition, child development

## Abstract

People often prefer similar others. The present study examined children’s use of three distinct similarity cues in their inferences about people’s social preferences. Participants were 144 children aged 4–8 years (49% female; 74% White). In a between-subject design, children observed a target actor demonstrate similar movements (movement condition), show the same liking/disliking of two toys (liking condition), or speak the same non-native language (language condition) as one of two actors (the similar actor) but not the other dissimilar actor. Children were then asked who the target liked to play with between the two actors. The results showed that children chose the similar actor in the movement condition, regardless of whether they explicitly identified the similar actor, whereas in the liking condition, they did so only when they could correctly identify the similar actor. In the language condition, however, the target and the similar actor speaking the same non-native language had no detectable impact on children’s answers. These findings are discussed in terms of how different types of similarity cues may shape children’s understanding of others’ social preferences, while also highlighting the need to consider differences in task demands and cue identifiability across conditions.

## 1. Introduction

Understanding others’ *social preferences* (e.g., their choices of social interaction partners) can help individuals map the broader social structure, predict future alliances, and navigate group dynamics effectively (Seyfarth & Cheney, 2013). One cue that individuals may rely on to predict others’ social preferences is captured by the homophily principle, whereby social connections are formed based on perceived similarities in various aspects, such as movements, expressing liking of certain things, and belonging to the same social group (McPherson et al., 2001). Indeed, extensive research demonstrates that adults leverage such similarity cues not only to choose their own interaction partners (e.g., Byrne, 1971; Chartrand & Bargh, 1999; Montoya et al., 2008; Rakić et al., 2011) but also to infer the social preferences of others (e.g., Curry & Dunbar, 2013; Lakens & Stel, 2011; Lickel et al., 2000). While similarity is widely recognized as a powerful predictor of social preference, it is not a unitary construct but depends on the features or dimensions along which entities are compared (Goodman, 1972; Tversky, 1977). This raises the question of how different types of similarity may impact social inferences, a question that is particularly relevant in childhood, when similarity-based social reasoning continues to develop and become more differentiated.

Similarity cues examined in developmental research generally fall into three distinct cue types, each of which has typically been examined with a separate theoretical focus. Behavioral similarity involves aligned body movements, such as imitation, which have been proposed to function as signals of social alignment, fostering feelings of social closeness among the involved actors themselves (Chartrand & Bargh, 1999; Lakin et al., 2003; Over & Carpenter, 2013). Psychological similarity involves shared likes and attitudes, which have been proposed to serve as observable signals of shared underlying psychological states or dispositions, such as common tastes or views about the world, thereby eliciting interpersonal attraction and preference (Byrne, 1971; Liberman et al., 2014; McPherson et al., 2001; Montoya et al., 2008). Shared group membership, often marked by features such as language, race, or gender, has been proposed to activate category-based representations supporting inductive generalizations about shared attributes and expectations of preferential affiliation among ingroup members (Kinzler et al., 2007; Liberman et al., 2017; Shutts, 2015; Tajfel & Turner, 1979). Guided by these theoretical views, developmental research has examined how children make use of each type of similarity cue in their social reasoning, typically focusing on one cue type at a time.

A substantial body of research indicates that infants and children themselves draw on these cue types when forming their own social preferences. Children prefer individuals who imitate or move synchronously with them (Agnetta & Rochat, 2004; Carpenter et al., 2013; Cirelli et al., 2014; Cirelli et al., 2016; Meltzoff, 1990; Over et al., 2013), those who share their preferences (Fawcett & Markson, 2010; Hamlin et al., 2013; Mahajan & Wynn, 2012), and those who belong to the same social group marked by language, race/ethnicity, or gender (Aboud, 2003; Howard et al., 2015; Kinzler et al., 2011; Kinzler et al., 2007, 2012; Kinzler et al., 2009; Kinzler & Spelke, 2011; LaFreniere et al., 1984; Shutts et al., 2009; Shutts et al., 2013; Spence & Imuta, 2020; see Shutts, 2015, for a review).

Beyond their own social preferences, children also draw on similarity cues to infer *others*’ social preferences. Infants and preschoolers have been found to use behavioral similarity (e.g., imitation or synchronous movement) and psychological similarity (e.g., food preferences) to predict others’ social preferences (Cirelli et al., 2018; Fawcett & Tunçgenç, 2017; Liberman et al., 2018, 2021; Liberman & Shaw, 2019; Over & Carpenter, 2015; Pesowski et al., 2023; Powell & Spelke, 2018). Regarding social similarity, prior work has shown that children draw third-party inferences from social group membership markers, expecting same-gender (Shutts et al., 2013) and same-race (Roberts et al., 2017) individuals to prefer one another. To our knowledge, however, no study has examined whether children use shared language to make analogous inferences. The only relevant study focused on infants’ predictions of interaction valence (positive or negative) based on language (Liberman et al., 2017). Specifically, after watching two speakers speak the same language (both English or both Spanish) or different languages (English and Spanish), English-learning infants at nine months expected positive interactions between the two English speakers but negative interactions between speakers of different languages. These expectations were not found with the two Spanish speakers, suggesting that infants’ language-based social predictions may depend in part on the presence of a native-language anchor. Building on this finding, in the present study, we examined whether older children would extend similar inferences to two non-native languages, without using a native language as an anchor. To extend prior research using movement as a cue to behavioral similarity and toy liking/disliking as a cue to psychological similarity for making social preference inferences (e.g., Fawcett & Tunçgenç, 2017; Liberman & Shaw, 2019; Pesowski et al., 2023; Powell & Spelke, 2018), we also included these two cues. Because similarity is not a unitary construct (Goodman, 1972; Tversky, 1977), the three cues examined here are not directly equivalent. Our aim was therefore not to determine which cue most strongly guides inference, but to examine each cue on its own terms and compare the conditions descriptively.

In addition, we aimed to address an often overlooked question concerning the cognitive processes underlying social preference inferences: specifically, the relationship between explicit identification of a similarity cue and the social inference drawn from it. This is because if children fail to infer a social preference based on similarity, the source of that failure is otherwise ambiguous. It remains untested whether the failure, if any, stems from an inability to retain the similarity information (e.g., a lack of explicit acknowledgement regarding who shares the cue) or a conceptual disconnect (e.g., successfully identifying the similarity but not viewing it as predictive of social preference). For example, children can successfully retain similarity information (e.g., matching shirt color) but choose not to use it for social prediction (Fawcett & Markson, 2010), reflecting such a disconnect. On the other hand, research with adults on the “chameleon effect” and related “social glue” accounts demonstrates that behavioral mimicry can foster affiliation without participants’ conscious recognition of the similarity itself (Chartrand & Bargh, 1999; Lakin et al., 2003). Although this work concerns first-person affiliation rather than third-party inference, it suggests that certain forms of similarity processing may not require explicit awareness of such information. Accordingly, the present study explored whether children’s successful identification of similarity information was linked to the social preference inferences they made across the three similarity cue types. A further reason to examine explicit identification is that the three cue types may differ in cognitive demand. If cues imposing greater demand more strongly require explicit identification, the link between identification and inference should itself differ across cues.

To this end, children aged 4 to 8 years were randomly assigned, in a between-subject design, to one of three conditions respectively featuring one similarity cue: movement, liking/disliking, or language. They first watched familiarization videos in which a target actor shared the specific similarity with one of the two actors (similar versus dissimilar actor). Importantly, the actors performed their demonstrations independently, a design intended to minimize social responsiveness between actors, such as imitation, conformity, or behavioral coordination, leaving shared similarity as the primary basis for inferring social preference. This design choice, however, may also have reduced the interactive context in which these cues ordinarily convey social meaning, an issue we will revisit in the Discussion. Children then answered a social preference question, a preference strength rating, and a similarity identification question. We focused on the 4- to 8-year range for three reasons. First, social reasoning continues to develop across this period, making it well suited for examining whether children’s reliance on distinct cue types changes with age. Second, because the present study posed questions and collected responses through spoken language, we selected an age range in which children can reliably complete such a forced-choice task. Third, this range overlaps with that of prior studies of children’s third-party social inferences (e.g., Liberman & Shaw, 2019; Over & Carpenter, 2015; Pesowski et al., 2023), facilitating comparison.

## 2. Materials and Methods

### 2.1. Participants

Participants were 144 children aged 4–8 years (73 males; range: 4.02–8.00 years; *M* = 5 years, 11 months, 26 days, *SD* = 1.21 years). Forty-eight children were randomly assigned to each of the three conditions: the movement condition (25 males; range: 4.03–8.00 years; *M* = 6 years, 0 months, 21 days, *SD* = 1.24), the liking condition (24 males; range: 4.26–7.90 years; *M* = 6 years, 1 month, 28 days, *SD* = 1.09), and the language condition (24 males; range: 4.02–7.95 years; *M* = 5 years, 8 months, 29 days, *SD* = 1.29). Although participants were randomly assigned to conditions, the mean age in the language condition was numerically lower than in the other two groups. A one-way ANOVA confirmed that this difference was not statistically significant, *F*(2,141) = 1.53, *p* = 0.22, indicating that age distribution was comparable across conditions.

The sample size was determined as follows. A priori analysis in G*Power 3.1 (Faul et al., 2007) for a one-way, three-condition between-subject ANOVA, using an effect size derived from prior work (Cohen’s *f* = 0.48; converted from Kinzler et al., 2009), with alpha = 0.05 and desired power = 0.90, indicated a minimum total *N* = 60 (*n* = 20 per condition). This minimum sample size is consistent with previous studies employing comparable forced-choice tasks, which reported reliable effects with approximately 16 to 24 children per between-subject cells (e.g., Afshordi, 2019; Over et al., 2013; Spence & Imuta, 2020). To increase sensitivity for detecting age-related changes across the 4- to 8-year range, to preserve full counterbalancing, and to maintain equal cell sizes, we set our target sample at *N* = 144 (*n* = 48 per condition). This larger sample also reduced our reliance on the effect size derived from the first-person paradigm of Kinzler et al. (2009), which may overestimate the effect expected for third-party inference. A sensitivity analysis in G*Power confirmed that this final sample provided adequate power (0.80, alpha = 0.05) to detect small-to-medium between-condition effects (*f* = 0.26) as well as within-condition deviations from chance. An additional 13 participants were tested but excluded from the final analysis due to technical difficulties (*n* = 3; see below), providing no response or persistently answering “I don’t know” (*n* = 5), failure to understand the social preference question (*n* = 1), experimenter error (*n* = 3), or failure to pass the Likert-scale training (*n* = 1; see below).

Data collection occurred either in-person at science museums in two Midwestern cities (*n* = 25) or online via the Lookit study platform (*n* = 119; Scott & Schulz, 2017). Participants were predominantly White/European American (73.6%). Additional racial/ethnic identities included Asian American (27.1%), Black or African American (9.0%), Hispanic/Latino (4.9%), Native Hawaiian or Pacific Islander (1.4%), American Indian or Alaska Native (0.7%), Middle Eastern (0.7%), and South American (0.7%); because racial/ethnic categories were not mutually exclusive, percentages sum to more than 100%. All participants spoke English as their primary language, and only monolingual English-speaking children were assigned to the language condition. The study was approved by the university’s Institutional Review Board, and written informed consent was obtained from a parent or legal guardian for each child.

### 2.2. Procedure

Children participated in either an in-person or online session. For in-person participation, parents or legal guardians completed a hard-copy consent form; for online participation they completed an electronic consent form via Qualtrics, before the session began. After obtaining informed consent, each child was tested individually by an experimenter. During in-person sessions, children sat next to the experimenter and watched the videos on a 14-inch laptop monitor, wearing a headset to minimize external noise. Legal guardians were instructed to sit apart from the child (typically behind them) to ensure they did not influence the child’s performance. During online sessions, children joined via Microsoft Teams and the experimenter shared their screen to present the videos and pose questions. Legal guardians were asked to remain nearby to assist with potential technical issues but were instructed to refrain from interacting with the child unless explicitly requested by the experimenter. All online participants were required to use a 13-inch or larger screen and were not permitted to use mobile phones. Participants and their legal guardians were instructed to report any technical issues that arose at the start of or during the session. In all but 3 cases these issues were resolved promptly to allow the session to continue. For those three participants, the session was terminated and their data excluded. Families received a $5 Amazon gift card for their participation, regardless of whether the session was completed.

**Figure A1:**
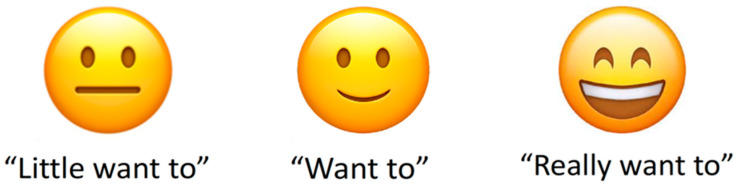
Three-Point Likert Scale.

To ensure children understood the response measure, a Likert-scale training phase was conducted before the testing session. The experimenter sequentially displayed images of a dog and a cat on the screen and asked children about their preference (e.g., “Do you like this dog/cat?”), to which they responded verbally with “yes” or “no”. Subsequently, the experimenter asked the child to rate the strength of their desire to play with that animal (e.g., “How much do you want to play with this dog/cat?”) using the 3-point scale (“Little Want To”, “Want To”, “Really Want To”; see Figure A1 in Appendix A). The experimenter monitored responses in real-time to assess whether the child understood the correspondence between their verbal preference and the scale. If children’s answers were inconsistent, the experimenter re-introduced the questions to clarify the use of the scale. If the child’s subsequent answers became consistent, the experimenter proceeded with the session. If the child’s answers remained inconsistent, the experimenter terminated the session and excluded the child’s data from the final dataset (*n* = 1).

**Figure 1:**
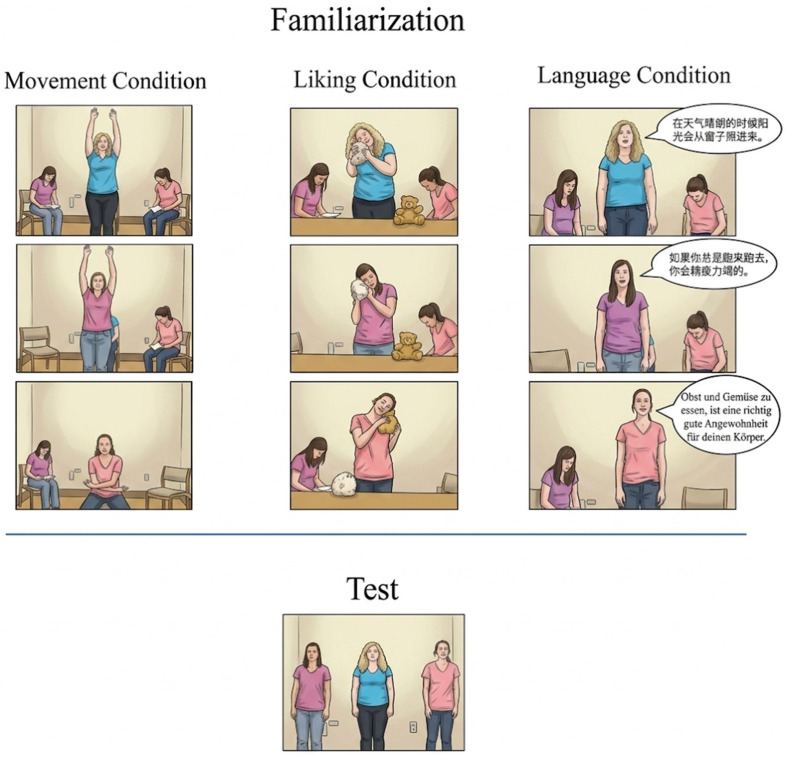
Example illustrations of the familiarization and test trials in the movement, liking, and language conditions. *Note.* In this example, the woman in the blue shirt is the target, the woman in the purple shirt is the similar actor, and the woman in the orange shirt is the dissimilar actor. During familiarization, the target and similar actor shared a specific movement (movement condition), the same liking/disliking for two toys (liking condition), or the same non-native language (language condition). The speech bubbles in the language condition show the original non-English stimuli used in the experiment; English translations of all sentences are provided in Table A1 (Appendix B). Actors spoke either Mandarin Chinese or German, and in this example the target and similar actor each spoke Mandarin Chinese while the dissimilar actor spoke German. Across participants, the spatial positions of the similar and dissimilar actors, their assigned roles, and the specific shared cues (specific action, toy liking/disliking, or language) were counterbalanced. After viewing the familiarization trials specific to their condition, children viewed the static test image and answered the test questions via verbal or pointing responses.

Following this training, the testing began. The experimenter introduced the video to children by saying, “Alright [child’s name], you and I are going to watch a fun show now! Are you ready? Let’s watch the show! Here we go!” Then, children in all conditions viewed four familiarization trials followed by a test trial featuring a static scene with the three actors during which they answered three test questions (see Figure 1). During familiarization, children watched two 40 s events twice (one event in each trial, hence four trials) that featured three actors including the target, the similar actor, and the dissimilar actor. The two events only differed in the order of actions by the similar and dissimilar actors. The centrally located target always acted first. In one event the similar actor acted next and was followed by the dissimilar actor, while in the other event this order was reversed. The target and similar actor performed the same movements in the movement condition, expressed the same liking/disliking towards two toys in the liking condition, and spoke the same non-native language in the language condition. The dissimilar actor, by contrast, performed different movements from the target, expressed the opposite liking/disliking for the toys, or spoke a different non-native language. Across participants, the positions of the similar and dissimilar actors on the left or right, their assigned roles regarding which actor was similar or dissimilar, and which of the two options in movements, liking/disliking, and languages was similar or dissimilar were counterbalanced. This resulted in eight possible presentation orders per condition. Children were randomly assigned to one of these orders. Because the present study focused on children’s inferences about social preference among three actors, the actors’ gender and race were held constant (all female and White) to ensure that these factors did not differentiate the similar and dissimilar actors.

At the start of each familiarization trial, the target sat in a chair positioned at the center along with the similar and dissimilar actors who sat in chairs on either the right or left of the screen while reading a book (see Figure 1). This setup ensured that while one actor performed her demonstration, the other two actors attended to their own books, thereby minimizing observable social responsiveness (e.g., mutual gaze or real-time imitation) among actors. This arrangement was maintained throughout each actor’s turn in all three conditions, such that the two non-performing actors remained oriented to their books for the entire duration of the performing actor’s demonstration.

#### 2.2.1. Movement Familiarization Trials

In the movement condition, once the trial started, the target walked to the center of the screen (3 s) and executed either Sequence A or Sequence B, which consisted of two 5 s movements. In Sequence A, the actor began by raising her right arm to shoulder height for 1 s and then did the same with her left arm for 1 s. She subsequently lifted both arms overhead into a raised position for 1 s, spread them horizontally at shoulder height for 1 s, and lowered them to her sides for 1 s. In Sequence B, the pattern began with the actor raising her right arm horizontally to the side for 1 s and then raising her left arm to match for 1 s. She crossed both arms downward while bending her knees outward for 1 s, straightened her knees while extending her arms horizontally for 1 s, and brought both arms back to her sides for 1 s. After the target completed the movements and returned to her seat (1 s), the similar actor also walked to the center (2 s) to execute the same movements as the target (10 s), returned (1 s), whereas the dissimilar actor walked to the center to perform a distinct movement sequence (13 s). The total duration of each familiarization event was 40 s.

#### 2.2.2. Liking Familiarization Trials

In this condition, the target walked to the center of the screen (4 s) and picked up one of the two toys, either a hedgehog or a lion stuffed animal. She said “Ew, I don’t like this” and returned the toy to its place (4 s). She subsequently picked up the other toy and lifted and hugged it while saying “Woo, I like this” before putting it back (4 s). She then returned to her chair and sat down to read (2 s). Following this, the similar and dissimilar actors each walked individually to the center of the screen for 3 s and demonstrated their liking/disliking. Their combined actions took 26 s. The total duration of each familiarization event was 40 s.

#### 2.2.3. Language Familiarization Trials

In this condition, the target walked to the center of the screen for 4 s, spoke in Mandarin Chinese or German for 8 s, and returned to her seat for 2 s. Next, the similar and dissimilar actors each walked individually to the center of the screen for 3 s, spoke either Chinese or German for 8 s, and then walked back for 2 s. The total duration of each familiarization event was 40 s. To facilitate discrimination, we selected languages from different rhythmic classes: Mandarin Chinese (syllable-timed) and German (stress-timed) (Mehler et al., 1996; Nazzi et al., 1998; Nazzi et al., 2000). This choice was motivated by evidence that infants as young as five months, and even newborns, can discriminate sentences from two unfamiliar languages when the languages differ in rhythmic class, but not when they belong to the same class (Nazzi et al., 1998; Nazzi et al., 2000). Selecting languages from different rhythmic classes therefore sought to render it relatively easier for children to distinguish the two non-native languages. To eliminate accent-related confounds, two Mandarin Chinese and two German native speakers dubbed all speech. Across familiarization trials, voice–actor pairings were held constant such that the target and the similar actor were dubbed by two native speakers of the target’s language with one speaker per actor. The dissimilar actor was dubbed by one of the two native speakers of the other language.

Children heard three sentences in each event and 12 sentences in total across the four familiarization trials. This consisted of either eight Mandarin Chinese and four German sentences or four Mandarin Chinese and eight German sentences, depending on the counterbalancing order. All the sentences within each video were unique and featured variation in the initial consonant across sentences (see Table A1 in Appendix B). This design ensured that children would make inferences based on language similarity rather than simple recognition of repeated sounds.

#### 2.2.4. Test Trial

After viewing the familiarization videos specific to their condition, all children saw a static image displaying the three actors in the same positions as in the familiarization trials (see Figure 1) and received three forced-choice questions in a fixed order. Children indicated their answer by either pointing to or verbally naming one of the two peripheral actors. Unlike in-person sessions where responses were directly observable, online sessions relied on camera visibility. Consequently, when camera angles obscured a child’s pointing behavior, the experimenter asked the child to clarify verbally. If the child did not provide a clarification (e.g., within approximately 120 s), the accompanying legal guardian was asked to report the child’s pointing direction.

First, children were asked a **social preference** question, “Between the woman in the purple/orange shirt and the woman in the orange/purple shirt, who do you think the woman in the blue shirt likes to play with?” Throughout the study, the experimenter always named the actor on the left side first. During in-person testing, the experimenter also pointed to the corresponding actor. During online sessions, instead of pointing, color-coded circles matching the actors’ shirt colors were superimposed on their still images as the question was read. If a child indicated that they did not understand the question or failed to answer, the question was repeated in a simplified form, “Does the woman in the blue shirt like to play with the woman in the purple/orange shirt or the woman in the orange/purple shirt?”. Children’s responses were coded as 1 for selection of the similar actor and 0 for the dissimilar actor. If a child refused to answer or repeatedly said “I don’t know,” their data were excluded from the final dataset (*n* = 5). One additional child was excluded because, despite being attentive, they failed to understand the social preference question even after multiple clarifications.

Second, children answered a **preference strength** question to assess the strength of social preference attributed to their choice. Children were asked to indicate the strength of the target’s desire to play with the selected actor. Response options were on a 3-point Likert scale (1 = “Little Want To”, 2 = “Want To”, 3 = “Really Want To”). Each response option on the scale was accompanied by a corresponding pictorial representation using emojis (see Figure A1 in Appendix A).

Last, children answered a **similarity identification** question to assess whether they recognized which actor shared a similarity with the target. This question was placed last to prevent prior explicit attention to the shared attribute from influencing children’s social preference judgment. The question asked, “Between the woman in the purple/orange shirt and the woman in the orange/purple shirt, who do you think moved the same way/liked the same toy/spoke the same language as the woman in the blue shirt?” When necessary, the question was repeated in a simplified form such as “Did she [points to or highlights the woman in the blue shirt] move the same way/like the same toy/speak the same language as the woman in the purple/orange shirt or the woman in the orange/purple shirt?”. Responses were recorded in the same manner and using the same coding scheme as the social preference question.

## 3. Results

The number of children in each condition (movement, liking, and language) who selected the similar actor on the social preference question and who correctly identified the similar actor on the similarity identification question is reported in Table 1; the full dataset is available at https://osf.io/mazjh/overview?view_only=95876d851c2f46d3a52cf77cdd0293bd (accessed on 7 May 2026). All analyses were conducted in R (version 4.4.2; R Core Team, 2024). Children’s responses to the two target questions, social preference and similarity identification, were binary, so binomial logistic regression served as the primary analysis for each, followed by two-tailed binomial tests comparing the proportion choosing the similar actor against chance (0.50), with a Bonferroni correction across the three conditions. Pairwise comparisons among conditions were Tukey-adjusted. To assess the strength of children’s inferred social preferences, responses on the 3-point Likert scale were analyzed with an ordinal logistic regression. Finally, to examine the relation between social preference and similarity identification, a logistic regression predicting social preference from condition, identification accuracy, and their interaction was conducted. In all regression models, the significance of main effects and interactions was assessed with chi-square tests. Age was included as a mean-centered continuous predictor in all models, preserving variation across the 4- to 8-year range. Having retained age as a continuous predictor, we used model-selection diagnostics to determine its functional form, which favored a linear specification. The linear age model (AIC = 192.0) fit better than a quadratic model (ΔAIC = +3.3; likelihood-ratio test: *χ*^2^(3) = 2.69, *p* = 0.44) and a cubic natural-spline model (ΔAIC = +8.6). A Hosmer–Lemeshow test indicated good overall fit for the linear model, *χ*^2^(8) = 5.58, *p* = 0.69, and visual inspection showed an approximately linear relation between age and the log-odds of choosing the similar actor. Preliminary analyses confirmed that demographic and procedural variables were not associated with children’s responses. Chi-square tests showed that sex (male, female), test modality (in-person, online), and the eight familiarization orders were not significantly associated with children’s responses to the social preference question (all *χ*^2^*s* < 9.50, all *ps* > 0.21). Subsequent analyses therefore did not include these factors as predictors.

### 3.1. Social Preference

**Figure 2:**
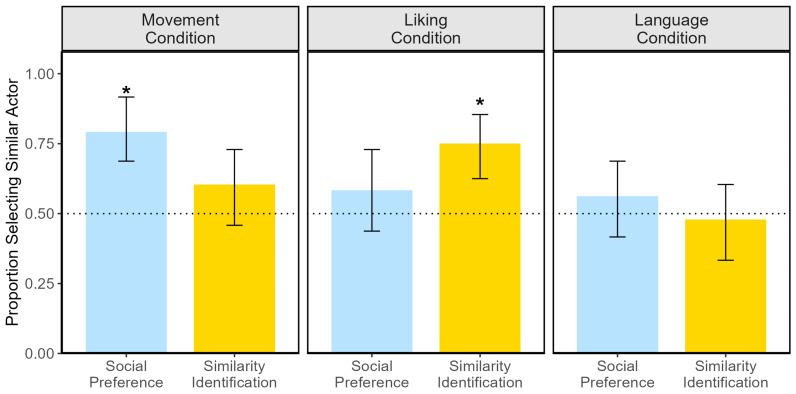
Proportion of children who selected the similar actor on the social preference question (blue) and on the similarity identification question (gold) in the movement, liking, and language conditions. *Note.* Bars show sample means. Error bars represent bootstrap 95% confidence intervals. The dotted line marks chance performance (0.50). Asterisks indicate proportions that differ significantly from chance (*p* < 0.05).

A binomial logistic regression predicting children’s responses to the social preference question (see Figure 2) from Condition (reference = movement), mean-centered Age, and their interaction revealed a significant main effect of Condition, *χ*^2^(2) = 6.89, *p* = 0.032, but no effect of Age, *χ*^2^(1) < 0.01, *p* = 0.989, and no Condition × Age interaction, *χ*^2^(2) = 0.04, *p* = 0.980. Relative to the movement condition, children were less likely to choose the similar actor in both the liking condition, *OR* = 0.37, 95% CI [0.14, 0.89], and the language condition, *OR* = 0.34, 95% CI [0.13, 0.84]. A Tukey-adjusted pairwise comparison indicated that the liking and language conditions did not differ from each other, *OR* = 1.06, *p* = 0.99.

Two-tailed binomial tests compared the proportion of children who chose the similar actor in each condition with chance (0.50). To account for the three planned comparisons, a Bonferroni correction was applied to the *p*-values. Only the movement condition differed significantly from chance: 38 of 48 children selected the similar actor (79.2%), 95% CI [0.65, 0.90], corrected *p* < 0.001. In contrast, proportions in the liking (28/48, 58.3%, 95% CI [0.43, 0.72]) and language conditions (27/48, 56.2%, 95% CI [0.41, 0.71]), did not differ from chance (corrected *ps* ≥ 0.93). Thus, children reliably selected the similar actor above chance only in the movement condition, whereas selection rates in both the liking and language conditions did not exceed chance.

To assess the strength of children’s inferred social preferences, we analyzed children’s responses to the preference strength question on the 3-point Likert scale regarding how much the target wanted to play with the selected actor (1 = “Little Want To” to 3 = “Really Want To”). An ordinal logistic regression with Condition (reference = movement), mean-centered Age, and their interaction revealed no significant main effect of Condition, *χ*^2^(2) = 1.36, *p* = 0.51, Age, *χ*^2^(1) = 1.26, *p* = 0.26, or Condition × Age interaction, *χ*^2^(2) = 0.45, *p* = 0.80. The predicted probability of choosing the highest strength response (“Really Want To”) did not differ significantly across the movement (47%), liking (48%), and language (38%) conditions. This indicates that while the proportion of children’s selections of the similar actor on the social preference question varied by condition, children’s perception of preference strength did not. In other words, children in the liking and language conditions attributed just as much preference strength to their choices as did children in the movement condition, even though their selections of the similar actor did not exceed chance.

Additional analysis confirmed that children’s attributed strength of social preference did not vary as a function of their choice of actor. There was no main effect of Choice Type, *χ*^2^(1) = 1.84, *p* = 0.17, nor a Choice Type × Condition interaction, *χ*^2^(2) = 3.14, *p* = 0.21. This indicates that children who chose the dissimilar actor were just as likely to report the highest level of preference strength (“Really Want To”) as those who chose the similar actor. For instance, in the liking condition, the predicted probability of reporting “Really Want To” was 57% for children choosing the dissimilar actor compared to 42% for children choosing the similar actor, though this difference was not statistically significant. This pattern indicates that children’s confidence in their choices did not vary with whether they selected the similar or dissimilar actor. In the liking and language conditions in particular, this pattern raises the possibility that dissimilar-actor choices reflected children’s own intuitions rather than uncertainty or random guessing, though this inference remains tentative given that the null finding does not preclude other interpretations.

### 3.2. Similarity Identification

A parallel binomial logistic regression model predicting children’s responses to the similarity identification question (see Figure 2) from Condition (reference = movement), mean-centered Age, and their interaction revealed significant main effects of Condition, *χ*^2^(2) = 6.36, *p* = 0.042, and Age, *χ*^2^(1) = 5.17, *p* = 0.023. The Condition × Age interaction was not significant, *χ*^2^(2) = 1.51, *p* = 0.47. Relative to the movement condition, the likelihood of correct identification was numerically higher in the liking condition (*OR* = 1.92, 95% CI [0.79, 4.81]) and lower in the language condition (*OR* = 0.64, 95% CI [0.27, 1.48]), though neither contrast was individually significant. Tukey-adjusted pairwise comparisons, however, showed that correct identification in the liking condition was significantly greater than in the language condition, *OR* = 3.02, *p* = 0.036; the movement condition did not differ from either of the other two conditions (adjusted *ps* ≥ 0.33). Regarding the significant age effect, each additional year of age increased the odds of correctly identifying the similar actor, *OR* = 1.77, 95% CI [1.08, 3.08].

Two-tailed binomial tests compared the proportion of children who identified the similar actor correctly in each condition against chance (0.50). As in the social preference analysis, a Bonferroni correction was applied to the *p*-values. Only the liking condition differed significantly from chance: 36 of 48 children responded correctly (75.0%), 95% CI [0.60, 0.86], corrected *p* = 0.002. Accuracy in the movement condition (29/48, 60.4%, 95% CI [0.45, 0.74]) and the language condition (23/48, 47.9%, 95% CI [0.33, 0.63]) did not differ from chance (corrected *ps* ≥ 0.58). These results indicate that children’s identification accuracy exceeded chance only in the liking condition, whereas accuracy in the movement and language conditions did not differ from chance.

### 3.3. Relation Between Social Preference and Similarity Identification

To examine whether correctly identifying which actor shared the target’s similarity predicted children’s social preference, we conducted a logistic regression that included Condition (reference = movement), similarity identification (identified correctly vs. failed to identify), mean-centered Age, and their interactions as predictors. The model revealed a main effect of Condition, *χ*^2^(2) = 11.85, *p* = 0.003, indicating that children in the movement condition were more likely to select the similar actor than those in the other conditions. Additionally, a significant Condition × Identification interaction was found, *χ*^2^(2) = 11.05, *p* = 0.004, suggesting that the relation between identification accuracy and social preference varied across conditions. Neither the main effect of Identification, *χ*^2^(1) = 0.62, *p* = 0.43, nor the effect of Age, *χ*^2^(1) = 0.29, *p* = 0.59, was significant.

**Figure 3:**
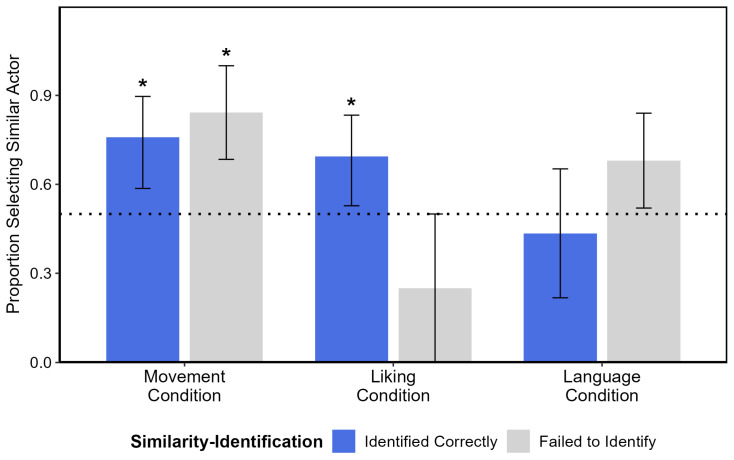
Proportion of children selecting the similar actor on the social preference question as a function of similarity identification accuracy (identified correctly vs. failed to identify) in the movement, liking, and language conditions. *Note.* Bars show sample means. Error bars represent bootstrap 95% confidence intervals. The dotted line marks chance performance (0.50). Asterisks indicate proportions that differ significantly from chance (*p* < 0.05).

Focusing on the effect of Identification within each condition (see Figure 3), in the movement condition, children chose the similar actor at a high rate regardless of whether they correctly identified the similar actor (*OR* = 0.55, *p* = 0.44). Notably, even children who failed to explicitly identify the similar actor showed a tendency to select her on the social preference question (Intercept *OR* = 5.54). In the liking condition, by contrast, correct identification was closely linked to social preference inference. Children who correctly identified the similar actor were significantly more likely to select her as the target’s social preference compared to those who did not (*OR* = 6.78, *p* = 0.012), indicating that explicit recognition of who liked/disliked the same toys as the target was closely associated with predicting the target’s social preference in this condition. In the language condition, identification accuracy was not significantly related to social preference inference: the odds of choosing the similar actor did not differ significantly between children who identified correctly and those who did not (*OR* = 0.35, *p* = 0.08).

To further examine this link, we conducted a sub-sample analysis including only children who correctly identified the similar actor. Within this sub-sample, children in the movement condition selected the similar actor significantly above chance (22/29, 75.9%, *p* = 0.008). Similarly, in the liking condition, the selection rate among children who identified correctly was 69.4% (25/36), also significantly above chance (*p* = 0.029). By contrast, children in the language condition did not select the similar actor above chance even when they correctly identified who spoke the same language as the target (10/23, 43.5%, *p* = 0.68).

These findings indicate that in the liking condition, successful identification of which actor shared the target’s liking/disliking predicted children’s similarity-based social preference inference. By contrast, in the movement condition, children selected the similar actor at high rates regardless of whether they explicitly identified the shared movement. In the language condition, even children who correctly identified which actor shared the target’s language did not reliably select that actor as the target’s social preference, suggesting that explicit identification of shared language alone was insufficient to guide social preference inference in this condition.

## 4. Discussion

The present study addressed two questions. First, we examined children’s social preference inferences across three distinct types of similarity cue: movement, liking/disliking, and language. Second, we investigated the extent to which these inferences depended on children’s explicit identification of the similar actor. The three cue types yielded different patterns of results. In the movement condition, children selected the similar actor at above-chance rates, and they did so regardless of whether they explicitly identified which actor had performed the same movement sequence as the target. In the liking condition, by contrast, children drew on shared liking/disliking of toys to infer social preference only when they successfully identified the actor who shared the target’s liking/disliking. In the language condition, children did not preferentially select the similar actor when inferring the target’s social preference, nor did they reliably identify which actor spoke the same non-native language as the target. Identification accuracy was also unrelated to social preference inference. Thus, in the language condition, neither shared non-native language nor its successful identification predicted children’s social preference inferences. Taken together, these results indicate that the three cue types differed in how children inferred social preference and in whether that inference depended on explicit identification. Regarding developmental trends, while the overall patterns of social preference inference remained relatively stable across the 4- to 8-year-old range, age played a significant role in the explicit identification of the similar actor. Specifically, older children were significantly more likely to correctly identify the similar actor across the three conditions, suggesting that while children’s use of similarity cues to make social inferences is present from early childhood, the cognitive process of explicitly identifying relevant social information continues to mature throughout this period.

The divergent pattern of results from the three conditions might be due to the different cognitive demands the similarity cues imposed, in terms of how readily the similarity itself could be perceived and how the cue could lead to a social preference inference. Shared movement may have been the most perceptually accessible cue, as the temporal alignment of body movements between two actors could be easily detected. Shared liking/disliking, by contrast, required children to track which actor expressed which valence toward each toy and then bind these actor-preference associations to the target’s preferences, a more demanding integrative process. Shared language posed an additional challenge, as both non-native languages were unfamiliar to children and there was no native-language anchor to facilitate the discrimination between them. In addition, the three cue types also differed in the form and amount of similarity evidence children received due to the nature of these cues: one movement sequence, expressed liking/disliking of two toys, and multiple spoken sentences. While temporal exposure was held constant across the conditions, as each familiarization event lasted 40 s and each actor’s demonstration was comparable in duration (approximately 8 to 10 s), it was admittedly difficult to equate cognitive and processing load across these qualitatively distinct cues. For this reason, the cross-condition comparisons described above should be interpreted with caution. In the following sections, we discuss the results of each condition in turn, focusing on their relevance to similar previous research.

In the movement condition, children reliably selected the similar actor as the target’s preferred play partner, regardless of whether they explicitly identified which actor had performed the same movement sequence as the target. Notably, identification accuracy itself did not exceed chance, indicating that children inferred the target’s social preference without reliably tracking which specific actor shared the movement, suggesting that the similarity could exert an influence on social preference inference in the absence of explicit identification. This raises the question of what the shared movements may have signaled to children. Because the actors did not interact during their demonstrations, these movements may not readily be interpreted as imitation or behavioral responsiveness between partners. We therefore speculate that the specific movements may have been perceived by children as resembling ritualized sequences, even without explicit recognition. Notably, the procedural structure may have reinforced this impression: each actor performed a novel movement sequence individually while the other actors attended to their books, creating a context in which the movements unfolded as discrete, formalized performances rather than ordinary, everyday behaviors. If so, the shared movements may have functioned as indicators of shared cultural knowledge, which has been shown to shape young children’s own social preferences (Soley & Spelke, 2016) as well as their inferences about social affiliation (Liberman et al., 2018). In addition to this cultural-knowledge interpretation, children may have construed the shared movements as evidence of shared abilities or dispositions. The present study cannot adjudicate among these explanations, and distinguishing them will require future work, for example, by employing movement sequences that vary in their ritual-like, culturally meaningful, or ability-relevant qualities.

In the liking condition, children’s social preference inference depended on whether they explicitly identified which actor shared the target’s liking/disliking. Although identification accuracy was generally above chance, children who correctly identified the similar actor were more likely to predict that the target would choose her as a play partner than those who did not. This pattern, however, diverges from previous research demonstrating that children can readily infer affiliation based on shared food or toy preferences (Liberman & Shaw, 2019; Pesowski et al., 2023). These divergent findings may reflect differences in experimental designs. In prior studies, preferences were often presented through clear narratives directly linking actors to shared liking (e.g., “This girl and this girl have the same favorite foods”; Liberman & Shaw, 2019) or by explicitly stating what they wanted (e.g., “Sara says she wants the popsicle,” “Becky says she wants the popsicle,” “Hazel says she wants a cupcake.”; Pesowski et al., 2023). In contrast, the current task required children to process 40 s sequences involving three actors. Crucially, unlike studies that provided explicit instructions on what to focus on before the task (e.g., “I want you to tell me which person she likes better”; Over & Carpenter, 2015), thereby allowing children to direct their attention strategically, children in the present study were not given any such prompt. Instead, they were simply asked to watch the events, requiring them to extract similarity patterns spontaneously without prior attentional guidance. This demand may have been further compounded by the specific procedural structure, wherein actors explicitly rejected an alternative toy (“Ew, I don’t like this”) before expressing their positive preference (“Woo, I like this”). Unlike the direct statements in previous work, this “disliking-first” sequence meant that children had to process both the initial rejection and the subsequent positive preference. This added complexity may have increased processing demands, potentially hindering similarity-based social preference inference when children failed to track which specific actor expressed which preference.

A further consideration concerns the absence of interaction between the actors, which was a feature of the design that attempted to minimize other social cues from similarity. Fourteen-month-old infants can infer affiliation from shared liking/disliking even when the actors do not interact, in the absence of other social cues (Liberman et al., 2021). The present findings extend this pattern to childhood, as shared liking/disliking supported social preference inference, at least among children who identified who shared the target’s preference.

In the language condition, children did not seem to draw similarity-based social preference inference from shared language. They neither reliably identified the similar actor nor preferentially selected her, regardless of identification accuracy. This pattern stands in contrast to prior research demonstrating that infants and children readily use language to guide their own social preferences, particularly when contrasting a native language with a non-native one (e.g., Kinzler et al., 2007; Kinzler et al., 2009; Liberman et al., 2017). As mentioned above, the language condition imposed particularly high demands on children because both languages were non-native and no familiar in-group language was available as an anchor. Even if children in the present study were capable of distinguishing the two non-native languages, they may have categorized both simply as “unfamiliar” and therefore not treated the distinction between them as a basis for inferring social preferences in the absence of a familiar in-group marker (Liberman et al., 2017). Alternatively, because children in the present study were not told in advance which feature to attend to, they may have failed to focus on the spoken language closely enough to detect the (dis)similarity. Importantly, even among children who correctly identified the similar actor, fewer than half chose her (10 of 23), suggesting that the null result may reflect properties of the task design. Specifically, because language ordinarily functions within interaction, removing this context may have weakened its value as a social cue. The use of dubbed voice-overs may have further added to these demands. Although our dubbing procedure held accent-related confounds constant, the voices were not produced by the actors themselves. As a result, vocal identity was decoupled from person identity, and language may not have been represented as a stable property of each individual, which could likewise have reduced its informativeness as a social cue. Future research could test whether shared non-native language supports social preference inference when it is embedded in social interaction and when both languages are produced by the actors themselves.

Aside from task and design features potentially contributing to the results, several further considerations specific to the language condition warrant attention. The first involves the role of environmental exposure. Because the current study did not measure participants’ exposure to linguistic diversity, the extent to which this factor influenced our results remains an open question. For instance, children might have found it unusual for White actors to speak Mandarin Chinese if they had learned that this was an East Asian language. Moreover, given that infants with greater exposure to linguistic diversity are more likely to expect cross-linguistic affiliation (e.g., expecting that people who speak different languages can share food preferences; Immel & Liberman, 2024), it is also possible that children in our study may have distinguished the languages but did not treat them as informative for predicting others’ social preferences, perhaps because they had learned that speaking a different language does not necessarily preclude social affiliation. Another consideration is that the mean age of children in the language condition was numerically younger than that of the other two groups, although the difference was not statistically significant. It thus remains possible that older children could use shared non-native language cues as a basis for social inference. Taken together, these considerations highlight the need for future research to examine whether shared language cues more reliably support social preference inference when accompanied by greater exposure to linguistic diversity and when tested in older age groups.

While the present study aimed to examine how children infer social preferences from different similarity cues, our investigation was limited to one exemplar of each of the three similarity cue types: a particular set of body movements, shared liking/disliking of toys, and two non-native languages. However, children may well display different patterns when other cue exemplars are used. For instance, with respect to behavioral similarity, the nature of the action may matter. While we used arbitrary movements, goal-directed actions (Kampis et al., 2013) or socially meaningful behaviors, such as musical synchrony (Cirelli et al., 2014), may carry greater social significance. Similarly, concerning psychological similarity, the type of preference may matter. While we used toys, sharing non-material preferences, such as music or stories, might drive stronger social preference inferences, as shared esthetic or cultural preferences often signal deeper compatibility than shared preferences for material objects (Boer et al., 2011; Rentfrow & Gosling, 2006). Finally, regarding social similarity, while the non-native languages used here did not guide social preference inference, other socially salient markers, such as gender and race, have been shown to guide both social categorization and social preferences in early childhood (e.g., Bigler & Liben, 2007; Dunham et al., 2011; Shutts et al., 2013; Roberts et al., 2017). Future research should broaden the repertoire of cues to include shared experiences (Afshordi, 2019), shared knowledge (Soley & Spelke, 2016), or incidental similarities (Liberman & Shaw, 2019), thereby yielding a more comprehensive account of how children’s social preference inferences unfold across different contexts.

Beyond expanding the range of cue exemplars, future work should also examine how children’s reliance on different similarity cues changes with age. In a companion study using closely matched stimuli with infants, similarity-based inference patterns differed from those observed here in 4- to 8-year-olds (Choi, n.d.). The two studies differed in test procedure, so this divergence should be interpreted cautiously. Still, it leaves open the possibility that how children use similarity cues in social preference inference changes with development. In the present study, social preference inference showed no clear association with age across the 4- to 8-year range. One possibility is that relevant change occurs mainly before this period, although the present data cannot establish this. With age, however, children may also become better able to integrate multiple sources of information when choosing interaction partners (e.g., Choi & Dunham, 2026), which could reduce the weight given to any single similarity cue. How children’s use of different similarity cues changes with age therefore warrants more systematic investigation across a broader developmental range. Such work could clarify how these inferences develop and which conditions make particular cues more or less informative.

Finally, several features of the present design constrain the generalizability of the findings. The actors were uniformly adult and female, and although these features were not expected to bear on the third-party inferences of interest, the extent to which the present findings generalize to peer actors and to actors of other genders remains a question for future research. Taken together, the present findings suggest that children’s social preference inferences may unfold differently depending on the type of similarity cue involved. However, because these conditions may also have differed in task demands and cue identifiability, the findings do not establish that the observed differences were caused by cue type alone. A fuller account will require examining these cues under more closely matched conditions to disentangle the effects of cue type from task-related factors, while also considering how children integrate multiple sources of social information when inferring others’ social preferences.

## Figures and Tables

**Table 1:** Number and percentage of children who selected the similar actor on the social preference question and correctly identified the similar actor on the similarity identification question, by condition.

Condition	*N*	Social Preference	Similarity Identification
Movement	48	38 (79.2%)	29 (60.4%)
Liking	48	28 (58.3%)	36 (75.0%)
Language	48	27 (56.2%)	23 (47.9%)

*Note.* Values represent the number of children, with percentages in parentheses. For the social preference question, values indicate the number of children who selected the actor who shared the similarity with the target. For the similarity identification question, values indicate the number of children who correctly identified the actor who shared the similarity.

## Data Availability

The raw data supporting the findings of this study are available on OSF at https://osf.io/mazjh/overview?view_only=95876d851c2f46d3a52cf77cdd0293bd (accessed on 7 May 2026).

## Appendix B

**Table A1:** All Sentences Used in Language Condition.

English	If you are always running around too much, you will be exhausted.
German	Wenn man zu viel herum rennt, ist man schnell erschöpft.
Chinese	如果你总是跑来跑去，你会精疲力竭的。
English	Eating vegetables and fruits is a really good habit for your body.
German	Obst und Gemüse zu essen, ist eine richtig gute Angewohnheit für deinen Körper.
Chinese	吃蔬菜和水果是一个对你身体很好的习惯。
English	Light shines through the window when it is really sunny outside.
German	Licht scheint durch das Fenster, wenn es draußen wirklich sonnig ist.
Chinese	在天气晴朗的时候阳光会从窗子照进来。
English	The leaves turn red and yellow when Autumn comes every year.
German	Jedes Jahr im Herbst färben sich die Blätter rot und gelb.
Chinese	每年秋天的时候叶子会变成红色和黄色。
English	The trees next to a bench in the park are big and green.
German	Die schönen Bäume neben der Bank in der Mitte des Parks sind groß und grün.
Chinese	公园里椅子边上的那棵树又大又绿。
English	He went to bed late yesterday but got up early to watch the sun rise.
German	Gestern ging er spät zu Bett, aber er stand früh auf, um den Sonnenaufgang zu sehen.
Chinese	他昨天晚上睡得很晚但是今天早上起早看日出。
English	Science has acquired an important place in modern western society.
German	Wissenschaft hat in der modernen westlichen Gesellschaft eine wichtige Rolle eingenommen.
Chinese	在现代西方社会里科学取得了重要地位。
English	The main library on campus is open every day from 8 a.m. to 6 p.m.
German	Die Hauptbibliothek auf dem Campus ist jeden Tag von acht bis sechs Uhr geöffnet.
Chinese	校园里的主图书馆每天从早上八点开到晚上六点。
